# Effect of Baseline Adjacent Segment Degeneration on Clinical Outcomes After Lumbar Fusion

**DOI:** 10.1177/21925682251318627

**Published:** 2025-02-01

**Authors:** Leevi A. Toivonen, Heikki Mäntymäki, Lorin M. Benneker, Hannu Kautiainen, Marko H. Neva

**Affiliations:** 1 Department of Orthopedics and Traumatology, Tampere University Hospital and Tampere University, Tampere, Finland; 2 Department of Neurosurgery, Tampere University Hospital and Tampere University, Tampere, Finland; 3 Orthopedic Department, 388063 Sonnenhofspital, Bern, Switzerland; 4 Primary Health Care Unit, 60650 Kuopio University Hospital, Kuopio, Finland; 5 3812 Folkhälsan Research Center, Helsinki, Finland

**Keywords:** disc degeneration, combined imaging score, adjacent segment disease, lumbar fusion, reoperation, oswestry disability index

## Abstract

**Study Design:**

Cohort Study.

**Objectives:**

End-stage degenerative segments above fusion levels have been associated with lower rates for surgical adjacent segment disease. We aimed to explore how this degeneration translates into patient reported outcomes.

**Methods:**

A consecutive series of lumbar fusion patients for degenerative pathologies were enrolled. Preoperative cranial adjacent segment degeneration status was determined using the Combined Imaging Score (CIS). Based on CIS, patients were trichotomized into mild (CIS <7), advanced (CIS 7-10), and end-stage (CIS >10) degeneration tertiles. In analysis, tertiles were adjusted for age, sex, and fusion length to control for potential confounders. The Oswestry Disability Index (ODI) was collected at baseline, 3 months, 1, 2, 5, and 10 years.

**Results:**

197 patients were included. Postoperative improvements were similar across all CIS tertiles at all time-points, except for the marginal 10-point difference at 2-years, in favor of higher degeneration. Despite similar changes, end-stage degeneration (CIS >10) implied marginally lower disability levels at baseline and throughout follow-up.

The 10-year all-cause mechanical reoperation rate was lowest in the third tertile (28%, 27%, 22%). Reoperation history was associated with greater disability among advanced degeneration (CIS 7-10); the ODI score difference 9 points, *P* = .009. Postoperative sagittal alignment was not reflected on physical performance.

**Conclusions:**

Preoperative adjacent segment degeneration status was only marginally reflected on 10-year disability outcomes. End-stage adjacent segment degeneration signaled lower reoperation risk and favorable functional outcomes. Our findings support the assumption that collapsed, end-stage degenerative segments without stenosis can be safely excluded from fusion constructs.

## Introduction

Lumbar spine fusion (LSF) is widely used to treat degenerative spine disorders. Optimizing LSF outcomes requires identification of relevant prognostic factors. Prior literature has evaluated the effects of disc degeneration status on LSF outcomes mostly inside the surgical target area with conflicting results.^1-3^ There are limited data on how degeneration adjacent to the fusion segment impacts outcomes. Surgeons may ponder if they can leave severely degenerative segments to the edge of fusion.

The Pfirrmann classification is the most used degeneration metric, but it has poor resolution with advanced degeneration.^4,5^ The Combined Imaging Score (CIS), which combines the most influential radiographic and magnetic resonance imaging parameters, is a more discriminative measure of degeneration.^6^ A recent paper showed that CIS-graded advanced degeneration at the cranial adjacent segment signaled an increased 12-year risk for reoperations after LSF, whereas end-stage degeneration appeared protective.^7^ Data are lacking how end-stage adjacent segment degeneration shapes patient-rated LSF outcomes. The role of adequately restored alignment on functional alignment is less clear with degenerative conditions compared to deformity.^8,9^

The objective of the present study was to evaluate how preoperative adjacent segment degeneration status is reflected on patient-reported outcomes through a 10-year follow-up after LSF. The secondary aim was to examine if reoperation status and postoperative sagittal alignment modify these effects.

## Methods

### Participants

This was a retrospective analysis of a prospectively collected institutional database. Patients were enrolled between 2008 and 2012 upon ethics committee approval and a written consent from all participants. Enrolled patients underwent LSF for degenerative indications (spinal stenosis with or without degenerative spondylolisthesis). Those with fusion to the thoracic spine, acute fracture, or tumor, or unavailable baseline imaging were excluded. The recruitment was described in detail elsewhere.^7^ All surgeries were open, posterolateral fusions with decompression of neural elements inside the fusion construct, and interbody spacers were used at the surgeon’s discretion.

### Data Collection

Standardized data collection included patient surveys at baseline and at 3 months, 1, 2, 5, and 10 years postsurgery. Surgeons recorded clinical and surgical data at the time of surgery. The primary outcome measure was the Oswestry Disability Index, which rates spine-related disability with a score ranging between 0-100 (a higher score for greater disability).^10,11^

The cranial adjacent segment degeneration status was determined by CIS from preoperative radiographs and MRIs by 2 raters as described elsewhere.^7^ The juxta-cranial adjacent segment was chosen to represent the degeneration status outside the surgical field as the caudal segment is not available with lumbosacral fusions. From postoperative lumbar standing radiographs, the following parameters were extracted by 2 authors: lumbar lordosis (LL), pelvic tilt (PT), pelvic incidence (PI), and PI-LL mismatch defined as PI-LL ≥9°.^9^

CIS is the sum of 6 subscores (Table 1) ranging between 0-17. CIS 7 has been reported as a threshold for severe degeneration.^6^ CIS tertiles (<7, −10, ≥10) stratified patients into mild, advanced, or end-stage degeneration cohorts.

**Table 1.** Combined Imaging Score (CIS) to Grade Intervertebral Disc Degeneration is the Sum of Six Sub-Scores.

Score	Radiographic Parameters			Magnetic Resonance Imaging Parameters		
	Height Loss (%)	Osteophytes	Intradiscal Calcification	T2-Signal Intensity	Modic Changes	Nucleus Shape
0	0-10	Margins rounded	No calcifications	Normal	Normal	Round/oval
1	10-20	Margins pointed	Rim calcification	Intermediate loss	Type I	Extension into inner annulus
2	20-30	<2 mm	Intranuclear calcification	Marked loss	Type II	Extension into outer annulus
3	>30	>2 mm	—	Absent signal	Type III	Extension beyond outer annulus

All mechanical reoperations (ie, reoperations for adjacent segment disease or instrumentation failure/pseudoarthrosis) within 10-years of surgery were collected by manual chart review. Also, potential dates of death were recorded.

### Statistics

Summary statistics were described using means with standard deviation (SD) or counts with percentages. The hypothesis of linearity was tested using the Cochran–Armitage test, analysis of variance, or logistic models with an appropriate contrast. The Kaplan-Meier method was applied to estimate the cumulative first mechanical reoperation rates. Incidence rates of all mechanical reoperations per 1000 person-years per baseline CIS were estimated using Poisson regression. Repeated measurements were analyzed using mixed-effects models with an unstructured covariance structure (ie, the Kenward–Roger method for calculating degrees of freedom). The possible non-linear relationship between CIS and ODI and reoperations were modeled using restricted cubic splines regression models (appropriate distribution and link function) with 3 knots at the 10th, 50th, and 90th percentiles; knot locations were based on Harrell-s recommended percentiles.^12^ We calculated the areas under the curve (AUC) with the trapezoidal method in terms of longitudinal ODI scores. AUC was divided by the total study time and results were depicted in time-weighted mean scores (Time-weighted AUC of ODI). Unadjusted correlations were calculated by the Spearman method, using Sidak-adjusted 95 percent confidence intervals (95% CI). All models allowed analyses of unbalanced datasets without imputation; therefore, we analyzed all available data with the full analysis set. Models were adjusted for age, sex, and fusion length when appropriate. Stata 16.1 (StataCorp LP, College Station, TX, USA) was used for the analysis.

## Results

### Patients

In total, 197 eligible patients were enrolled (Table 2). All participants had spinal stenosis, 80% accompanied with degenerative spondylolisthesis. Fusions were concentrated to the lower lumbar spine. Cobb angle of the juxta-cranial adjacent disc level decreased with increased degeneration, but otherwise postoperative sagittal alignment did not differ across CIS tertiles. During follow-up, 35 (18%) patients died.

**Table 2.** Patient Characteristics and Baseline Clinical Data.

	Combined Imaging Score (CIS) Tertiles			P for Linearity
	≤6	7-10	>10	
	N = 54	N = 81	N = 62	
Women, n (%)	45 (83)	65 (80)	42 (68)	.043
Age, mean (SD)	59 (11)	69 (8)	68 (9)	<.001
Smoking, n (%)	6 (11)	3 (4)	1 (2)	.024
BMI, mean (SD)	27.8 (5.0)	28.6 (4.3)	29.4 (4.0)	.050
Physical activity, hour/week, mean (SD)	5.6 (6.1)	6.3 (7.1)	8.4 (9.8)	.064
Education years, mean (SD)	12.0 (3.8)	10.0 (3.1)	11.7 (4.4)	.85
Duration of spinal complaint, mean (SD)	9.2 (9.1)	14.7 (13.9)	15.8 (15.2)	.007
VAS, mean (SD)				
Back pain	60 (26)	62 (29)	64 (22)	.37
Leg pain	6 8 (22)	70 (24)	65 (23)	.46
ODI, mean (SD)	47 (15)	46 (15)	43 (16)	.24
Self-reported comorbidities, n (%)				
Cardiovascular	26 (50)	46 (65)	39 (67)	.069
Rheumatoid	4 (8)	6 (8)	4 (7)	.87
Psychiatric	3 (6)	2 (3)	0 (0)	.066
Pulmonary	1 (2)	8 (11)	1 (2)	.89
Diabetes	5 (10)	10 (14)	8 (14)	.52
Neurological	2 (4)	1 (1)	1 (2)	.46
Indication for surgery, n (%)				.060
Spinal stenosis with DS	45 (83)	67 (83)	43 (69)	
Spinal stenosis without DS	9 (17)	14 (17)	19 (31)	
Juxtacranial adjacent level, n (%)				<.001
T12/L1	0 (0)	0 (0)	2 (3)	
L1/2	2 (4)	10 (12)	22 (35)	
L2/3	24 (44)	34 (42)	24 (39)	
L3/4	27 (50)	35 (43)	12 (19)	
L4/5	1 (2)	2 (2)	2 (3)	
Fusion length, levels				<.001
1-2	48 (89)	57 (70)	25 (40)	
≥3	6 (11)	24 (30)	37 (60)	
Postoperative sagittal alignment				
Juxta-cranial segmental angle, mean ° (SD)	12 (4)	11 (3)	6 (4)	<.001
PI-LL mismatch, n (%)	18 (33)	25 (31)	29 (47)	.12
PT, mean ° (SD)	20 (8)	21 (6)	21 (8)	.34
PI, mean ° (SD)	57 (11)	57 (10)	55 (10)	.23

SD, standard deviation; BMI, body mass index; VAS, visual analogue scale; ODI, Oswestry disability index; DS, degenerative spondylolisthesis; PI-LL mismatch, pelvic incidence minus lumbar lordosis ≥9°; PT, pelvic tilt; PI, pelvic incidence.

**Figure 1.**
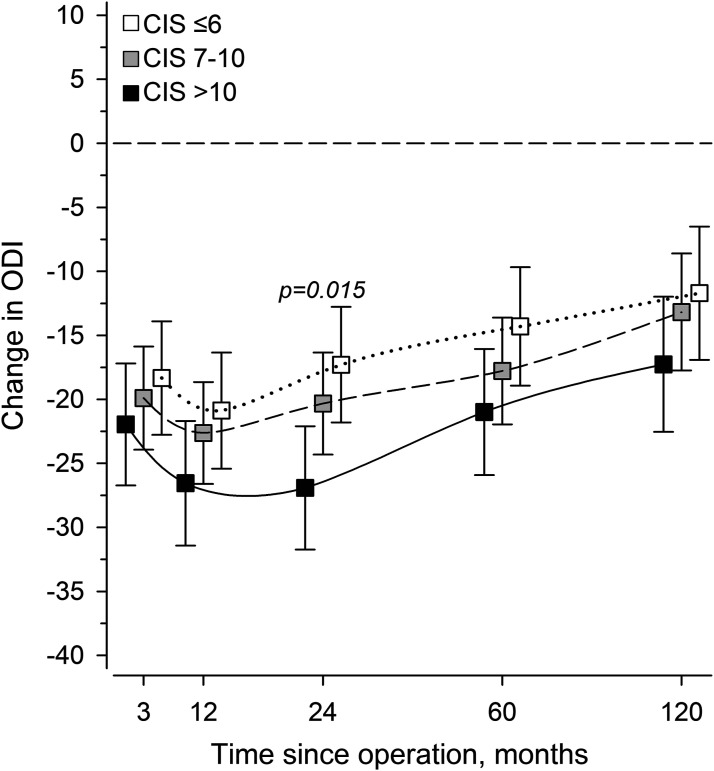
The Oswestry disability index (ODI) change scores after surgery per Combined imaging score (CIS) tertiles, adjusted for age, sex, and fusion length. The horizontal dotted line represents baseline.

All CIS tertiles gained significant patient-reported benefits from surgery at all time-points (Figure 1). After controlling for age, sex, and fusion length, ODI changes were similar in all CIS tertiles except for the marginally greater 2-year improvement in those with highest degeneration; the mean difference, 10 (SD 4 to 16) points, *P* = .015. An insignificant trend of greater improvement with more advanced degeneration was seen throughout follow-up.

**Figure 2.**
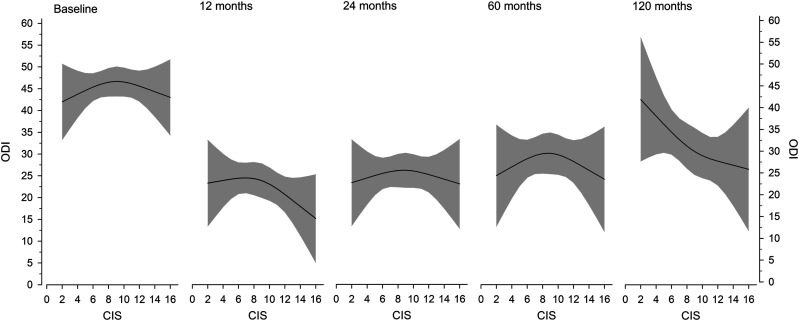
Adjusted (age, sex, fusion length), 3-knot restricted model predicting the Oswestry disability index (ODI) at baseline and follow-up per baseline degeneration status (combined imaging score, CIS). Gray areas represent the 95% confidence intervals.

Baseline CIS was reflected on patient-reported outcomes so that ODI was highest with intermediate degeneration (CIS 7-10) (Figure 2). At 10-years, mild baseline degeneration (CIS <7) signaled worse patient-reported outcomes, while end-stage degeneration (CIS >10) was associated with superior outcomes (Figure 2).

**Figure 3.**
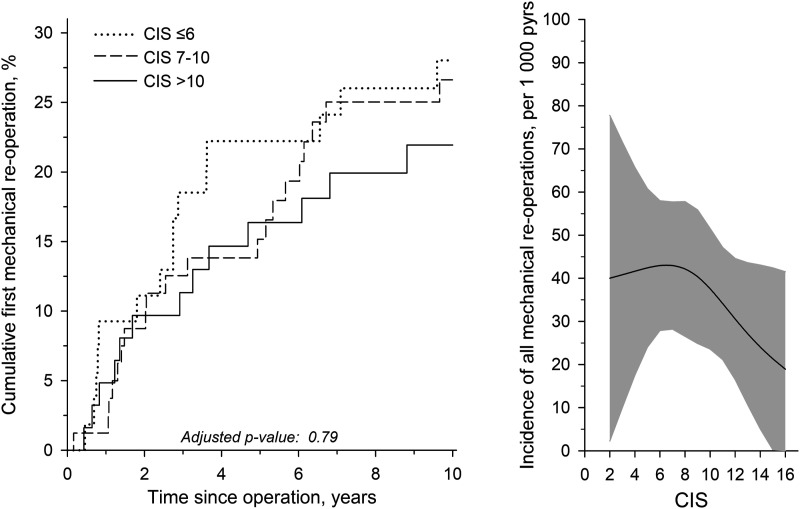
Left, cumulative first mechanical reoperation rates in combined imaging score (CIS) tertiles. Right, incidence rates of all mechanical reoperations per 1000 person-years per baseline CIS. The curve was derived from a 3-knot restricted cubic splines poisson regression model. The model was adjusted for age, sex, and fusion length. The gray area represents the 95% confidence intervals.

By 10-years, 25 (13%) patients underwent one reoperation for cranial adjacent segment disease, 7 (4%) for caudal adjacent segment disease, 7 (4%) for instrumentation failure, and 9 (5%) underwent 2 or 3 mechanical reoperations. Cumulative first reoperation rates are presented in Figure 3, left. The 10-year reoperation rates for CIS tertiles were: 28% with CIS <7; 27% with CIS 7-10; and 22% with CIS >10. Incidence rates of all mechanical reoperations per CIS are depicted in Figure 3, right.

**Figure 4.**
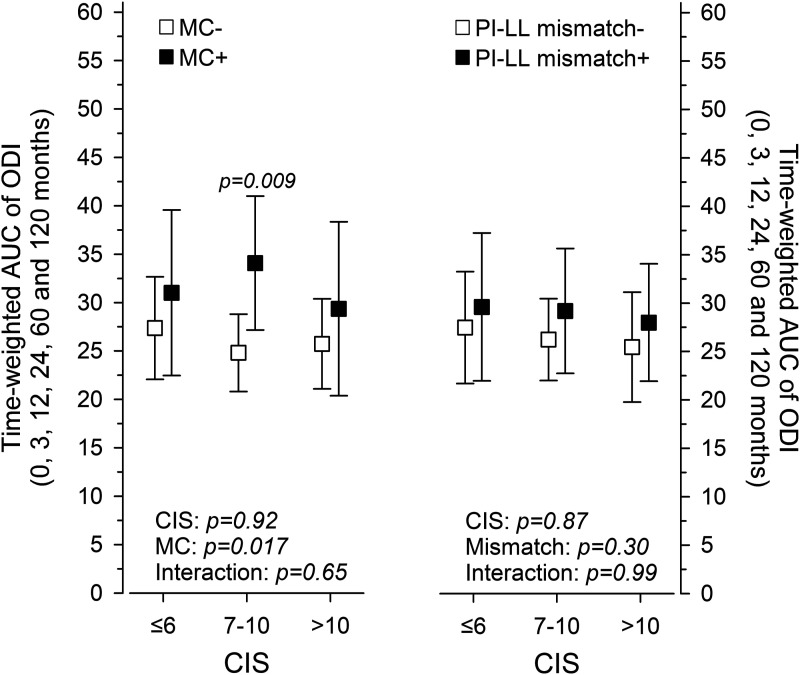
Left, time-weighted AUC of ODI in patients with positive (black) and negative (white) mechanical reoperation status (MC) per combined imaging score (CIS) tertiles. Right, time-weighted AUC of ODI in patients with (black) and without (white) PI-LL mismatch (pelvic incidence minus lumbar lordosis ≥9°). Whiskers show the 95% confidence intervals.

Follow-up ODI scores were summed into time-weighted mean scores, AUC of ODI, to represent the long-term performance of patients. Reoperation status discriminated clinical outcomes with the intermediate CIS tertile (Figure 4). There, positive reoperation history was associated with worse outcomes (*P* = .009). Postoperative PI-LL mismatch was not reflected in clinical outcomes by AUC of ODI (Figure 4). No single item of ODI appeared to be associated with baseline CIS level (Table 3).

**Table 3.** Spearman Correlations Between the Time-Weighted AUC of the Oswestry Disability Index (ODI) and Baseline Combined Imaging Score (CIS) of the Adjacent Segment.

Time-Weighted AUC_0-120 month_	r (95% CI^a^)
ODI total score	.05 (−.15 to .25)
ODI items	
1. Pain intensity	−.02 (−.23 to .18)
2. Personal care	.10 (−.11 to .29)
3. Lifting	.14 (−.07 to .33)
4. Walking	.14 (−.06 to .33)
5. Sitting	−.04 (−.24 to .17)
6. Standing	.03 (−.18 to .23)
7. Sleeping	−.03 (−.23 to .18)
8. Sex life	−.02 (−.23 to .18)
9. Social life	.00 (−.20 to .20)
10. Traveling	.03 (−.17 to .24)

^a^Sidak multiplicity corrected confidence intervals.

## Discussion

This study showed that preoperative adjacent segment degeneration had in essence no interference to postsurgical improvements. End-stage degeneration signaled lower disability at baseline and throughout follow-up. Intermediate degeneration was associated with increased reoperations and inferior outcomes in conjunction.

### Aligned Improvements

Previous studies have demonstrated little or no effect of disc degeneration status on surgical outcomes. Most of those studies have focused on degeneration status inside the surgical target region.^1,2^ Few studies have reported effects of adjacent segment degeneration on clinical outcomes. In their small series of 25 patients, Throckmorton et al reported similar outcomes after LSF for degenerative indications with vs without preexisting adjacent segment degeneration.^13^ Likewise, Okuda et al found no correlation with preexisting adjacent segment degeneration and clinical outcomes after L4-5 fusion.^14^ Conaway et al studied the effects of caudal adjacent segment degeneration on L4-5 fusion outcomes.^15^ They found no effect of Pfirrmann grade on disability outcomes, Yet, decreased anterior disc height at L5-S1 did signal superior back pain outcomes. Our findings of aligned improvements regardless of baseline degeneration status are in line with prior reports. It is compelling to suggest that disc degeneration status outside the surgical target is secondary to benefits from eliminating instability and achieving neural decompression.

### End-Stage Degeneration Signaling Better Performance

Despite aligned ODI change scores, end-stage degeneration was associated with modestly superior performance (lower ODI levels) in comparison to intermediate degeneration both at baseline and throughout follow-up. This is at odds with the reported weak positive correlation between disc degeneration status and disability.^16,17^ However, the natural course of spinal degeneration may result in collapsed motion segments and pain resolution.^18^ Apparently, this degenerative cascade may play a role in observed lower disability levels with end-stage degeneration both at baseline and at follow-up, considering that no stenotic segments were left next to the fusion.

There was a trend toward longer fusions reaching more cranially with higher CIS, reflecting the overall more advanced degeneration status. The observed lower segmental disc angle at juxta-cranial to fusion may be secondary to both more advanced degeneration and more cranial location. It is theoretically possible that even in the lumbar spine, certain upper instrumented levels may fare better than others. This study is not capable to answer to this question. Analyzes were adjusted for fusion length (which is associated with the upper instrumented vertebra) to reduce this possible bias.

### Revision Surgeries

Previously, the CIS mid-range was attributed to increased revisions for cranial ASD, while the upper tertile signaled reduced ASD risk.^7^ The present study collected all-cause mechanical reoperations, namely also pseudoarthrosis and caudal ASD for whom juxta-cranial degeneration status apparently is less relevant. However, undergoing revision surgery obviously has repercussions on patient-reported outcomes. Positive reoperation history discriminated the long-term outcomes only with advanced degeneration status (the intermediate CIS tertile). The need for reoperations signaled erosion of LSF achievements mostly in that subgroup. Our approach masked potential regains after revisions, that Maruenda et al reported in their retrospective study, where positive reoperation status signaled superior outcomes at 10-years and beyond.^19^ End-stage degeneration concurred with lower reoperation risk and favorable patient-reported outcomes. These findings deserve to be weighted in treatment decisions concerning the planned fusion levels.

### Sagittal Alignment

Balance disorders especially in the lumbar spine are poorly tolerated in deformity populations.^8,20^ The benefit of balance restoration with degenerative pathologies remains more ambiguous.^9,21^ This study included only degenerative patients with predominantly localized pathology and no coronal deformity. The observed insignificant trend toward more prevalent PI-LL mismatch with advancing degeneration status was not reflected on patient-reported outcomes. It is possible that this population had to some extent gradually adapted to the loss of lordosis. Moreover, the detriment of balance disorder is presumptively linked to the degree of balance disorder. Postoperative PT, reflecting the degree of pelvic compensation, was similar across CIS tertiles. These results indicate balance disorders have not confounded the current findings on CIS-ODI associations.

### Strengths and Limitations

Prospectively collected data, validated PROM instruments, and a validated, high-resolution degeneration measure are strengths of the present study. Increased lengths of follow-up may inherently add external confounding to outcome evaluation as other comorbidities increasingly cause functional restrictions. PROM collection scheduling based on the index surgery prevented more-detailed analysis of reoperations. Postoperative radiographs predominantly covered only the lumbar spine, thus limiting total balance evaluation. This drawback remained marginal given that the patients were not deformity patients. The consistently observed, although modest, positive influence of end-stage degeneration on outcomes corroborates the idea that end-stage degenerative segments fare without fusion.

## Conclusions

Preoperative adjacent segment degeneration status did not modify 10-year disability outcomes. End-stage adjacent segment degeneration signaled lower reoperation risk and favorable functional outcomes. Our findings support the assumption that collapsed, end-stage degenerative segments without stenosis can be safely excluded from fusion constructs.
